# Extracts from By-Products of the Fruit and Vegetable Industry as Ingredients Improving the Properties of Cleansing Gels

**DOI:** 10.3390/molecules30244687

**Published:** 2025-12-07

**Authors:** Agata Blicharz-Kania, Magdalena Iwanek, Anna Pecyna

**Affiliations:** 1 Department of Biological Bases of Food and Feed Technologies, University of Life Sciences in Lublin, Głęboka 28, 20-612 Lublin, Poland; 2 Department of Technology Fundamentals, University of Life Sciences in Lublin, Głęboka 28, 20-612 Lublin, Poland; anna.pecyna@up.edu.pl

**Keywords:** loan chemical extraction, antioxidants, cleansing gels, sustainable development

## Abstract

This study aimed to evaluate the effect of adding extracts obtained from by-products on the physicochemical and functional properties of cleansing gels. Micellar extraction (2% decyl glucoside solution in water) was performed on secondary raw materials: banana peel (BP), pomegranate peel (PP), tomato pomace (TP), and grape pomace (GP). The extracts were analyzed for soluble substances and active compounds (polyphenols, carotenoids, and vitamin C). Cleansing gels containing plant extracts were also prepared and evaluated for their color and physicochemical and functional properties. The extracts contained natural polyphenols (10.99–16.54 mg·100 mL^−1^), carotenoids (1.391–2.402 mg·mL^−1^), and vitamin C (0.651–1.529 mg·100 mL^−1^). The extract-enriched gels showed altered color (lower brightness, greater redness and yellowness), enhanced foaming properties, and modified viscosity (402.9–416.8 mPA for BP and GP; lower for PP and TP). The pH of the gels ranged from 5.391 to 5.917, which is within the physiological range of human skin. Dissolution times were reduced by up to 60% compared to the control, with PP extract producing the shortest time of 15.7 min. These results indicate that plant by-product extracts can improve both the functional performance and skin compatibility of cleaning gels.

## 1. Introduction

Shower gels are one of the most commonly used liquid body-wash products [1]. Consumers expect shower gels to provide effective cleansing without degreasing the skin [2]. Increasingly, in addition to their cleansing properties, these products are also required to moisturize, soften, and pleasantly fragrance the skin [3]. To meet consumer expectations, cleansing products incorporate detergents as well as ingredients that ensure product stability, caring properties, and high sensory quality. Moisturizing cleansing gels should contain active ingredients that soothe the skin, rebuild the lipid barrier of the stratum corneum, and contain humectants and skin nourishing substances. These properties can also be achieved by enriching traditional cleansing formulas with natural cosmetic ingredients, including plant-based ingredients, such as fruit and vegetable by-products.

Raw materials obtained from fruit and vegetable processing can be considered a rich source of chemical substances with significant skin care potential, e.g., carotenoids and polyphenols [4]. These compounds exhibit a broad spectrum of action on the skin and can be incorporated into cosmetic products, especially those with moisturizing, anti-aging, and antimicrobial properties. Raw materials extracted from agri-food waste are often richer in bioactive compounds than their counterparts extracted from fresh raw materials. For example, waste from the olive industry can be a richer source of polyphenols than olive oil itself, and tomato peel contains almost five times more lycopene than tomato flesh [5,6]. According to Bharadvaja’s [7] research, long-term use of cosmetics containing polyphenols can be effective in reducing wrinkles, discolorations, freckles, and actinic keratosis (skin lesions caused by excessive exposure to UV radiation).

Banana peel may be a very promising secondary raw material. Annual banana production exceeds 100 million tons, with the peel accounting for about 35% of the weight of the entire fruit [8,9]. The peel has traditionally been used as a medicinal material to treat various ailments, such as burns, anemia, diarrhea, ulcers, inflammation, diabetes, cough, snakebite, and excessive menstruation [10]. In recent years, experiments have been conducted to identify the substances responsible for the medicinal effects of banana by-products. The active compounds present in banana peel extract include glycosides, alkaloids, saponins, carotenoids, biogenic amines, tannins, anthocyanins, epicatechin, catechin, flavonoids, phytate, and 18 different types of amino acids. Banana peel extract also contains minerals, such as iron, calcium, sodium, phosphorus, and magnesium. In addition, it is a rich source of omega-3 and omega-6 polyunsaturated fatty acids, namely linoleic and linolenic acids. The extract has antioxidant and anti-inflammatory properties and exerts firming, anti-aging, and skin-brightening effects. The antibacterial properties of ethanolic banana peel extract against both Gram-positive and Gram-negative bacteria, such as *Salmonella typhi*, *Bacillus subtilis*, and *Staphylococcus aureus*, have also been confirmed [11,12,13]. Furthermore, in the cosmetics industry, banana starch can be used as a thickening and gelling agent, as well as a stabilizer in emulsions. It also improves the smoothness of emulsions during application to the skin and can replace talc in face and body powder formulations [8].

Pomegranate (*Punica granatum* L.) residues also appear to be interesting from the perspective of their use in cosmetic formulations. The antioxidant potential of pomegranate is attributed to polyphenols, such as punicalins, punicalagins, gallstones, and ellagic acid. Polyphenols are the dominant compounds in pomegranate peel and are composed mainly of hydrolyzable tannins. Extracts from pomegranate peel or seeds have shown remarkable antioxidant and antibacterial properties, with high free radical scavenging capacity [14,15,16,17,18]. Pomegranate by-product extract provided significant protection against UVB-induced damage in studies conducted on cultured human fibroblasts. It promoted proliferation and procollagen synthesis and reduced MMP-1 (also known as interstitial collagenase) production in human skin fibroblasts [19,20,21]. In Chinese medicine, pomegranate peel (*Pericarpium Granati*) has been used for centuries in skin care and in the treatment of mucous membrane disorders, among other things. As confirmed by the Pharmacopoeia of the People’s Republic of China (2020), it is attributed with astringent, anti-diarrhoeal, haemostatic and antihelminthic properties [22]. Contemporary scientific research remains consistent with this traditional knowledge. Both Chinese medical theory and network pharmacology analyses point to pomegranate peel as a potential anti-inflammatory and anti-cancer agent [22]. Recent phytochemical studies have shown that granatin B and punicalagin are among the most potent antioxidants present in pomegranate peels, as confirmed by LC–MS/MS analyses and antioxidant activity studies [23]. These results provide a mechanistic explanation for the traditional uses of pomegranate peels, including those related to the protection of the skin and mucous membranes. Tomato pomace can be another valuable secondary cosmetic raw material. Globally, the annual production of fresh tomatoes reaches approximately 160 million tons, and nearly 40 million tons of tomatoes are processed into tomato products: tomato paste, ketchup, sauces, and canned tomatoes [24]. Tomato pomace, a by-product of tomato processing, consists of peels (approx. 50%), seeds (approx. 40%), and a small amount of pulp. It contains valuable compounds, such as fiber, proteins, carotenoids, sugars, pectins, fat, minerals, and antioxidants [25,26]. Lycopene, the main carotenoid found in tomatoes, is a powerful antioxidant. It protects the skin from UV damage by reducing epidermal ornithine decarboxylase activity, maintaining normal cell proliferation, and preventing DNA damage caused by blocking apoptosis (specifically, by blocking the caspase-3 apoptotic pathway). Topical application of lycopene in a microemulsion is a convenient way to increase its delivery to the skin and antioxidant activity in the tissue. To increase the efficiency of lycopene extraction, tomato peels are enzymatically pretreated with pectinolytic enzyme preparations, followed by surfactant-assisted extraction [24,27,28,29].

The most extensively studied by-product in terms of skincare benefits is grape residue. Grape pomace is the main residue associated with the wine industry. It accounts for 20 to 25% of the initial grape weight and is composed of 25% seeds, 25% stems, and 50% skins [30,31,32]. The active substances present in grape pomace include unsaturated fatty acids, dietary fiber, vitamins, and natural antioxidants [33]. The latter can be divided into various groups: phenolic acids, flavonoids, tannins, lignans and neolignans, stilbenes, coumarins, and phenylethanol derivatives. Grape pomace polyphenol extracts have demonstrated primarily antioxidant, anti-aging, anti-hyperpigmentation, and UV-protective effects in in vitro tests [34,35]. In addition, grape by-products are also the richest source of resveratrol—a type of natural phenol with excellent antioxidant properties. Resveratrol, found in the skin of red grapes, has been shown to have protective effects against inflammatory skin diseases, e.g., sarcoidosis. It reduces MMP-9 expression, thereby protecting collagen from UV radiation, and protects human keratinocytes from damage caused by oxidative stress [36,37]. Conventional methods for extracting bioactive compounds from fruit and vegetable waste, such as Soxhlet extraction, maceration, and steam distillation, typically involve the use of water-alcohol mixtures as solvents and are time-consuming. They typically require prolonged heating, which promotes the degradation of the desired compounds. This emphasizes the need for more innovative extraction techniques [38,39]. An alternative method is cloud-point extraction (CPE), also known as micellar extraction, the liquid concentration technique, or micelle-mediated extraction. CPE involves the use of surface-active compounds, or surfactants. These substances consist of both hydrophilic and hydrophobic moieties. At low concentrations, surfactant molecules exist in aqueous solutions in a monomeric form and sometimes also as dimers and trimers. As their concentration increases, surfactant monomers accumulate, forming colloidal-sized aggregates, or micelles, above a limit called the critical micellization concentration (CMC). Regardless of their shape and size, surfactant molecules orient their hydrophobic portions toward the center of the aggregate formation. Hydrophobic (poorly water-soluble) substances can be dissolved within these micelles [40,41].

Ultrasound-assisted extraction is also an environmentally friendly technique for recovering valuable components from polysaccharides, pectins, essential oils, and antioxidants [42]. In this method, acoustic waves generated in the solvent induce cavitation bubbles that burst on the surface of the raw material, causing disruptions in the cell wall. These disruptions facilitate the release of active compounds into the solvent. This technique is optimized by controlling solvent concentrations, solvent-to-sample ratio, extraction time, temperature, frequency, and power [43,44].

The main aim of the study was to evaluate changes in the physicochemical and functional properties of shower gels caused by the addition of extracts obtained from by-products through chemical extraction using borrowed substances. In the present experiment, the extraction process was employed as one of the stages of shower gel production. The extraction medium consisted of raw materials used as ingredients for the cosmetic product. The solution was prepared to obtain aggregates (micelles) in the bulk phase, facilitating effective leaching of active ingredients from secondary raw materials, as in CPE. This method, referred to as “loan chemical extraction” (LCE), has been described in the literature as an effective and sustainable extraction technique [45,46,47]. LCE allows the efficient extraction of bioactive compounds, preserving their biological activity, with minimal use of additional chemical solvents. Furthermore, the resulting extract can be directly incorporated into the final cosmetic formulation, simplifying the production process and increasing its sustainability. This method is particularly beneficial for ingredients that are temperature-sensitive and sensitive to aggressive chemicals, enabling their effective integration into the final cosmetic product [45,46,47].

## 2. Results and Discussion

### 2.1. Content of Soluble Solids and Antioxidants in Extracts

In the extracts from the waste raw materials, the differences in soluble substance content ranged from 2.13 to 2.47 °Brix (Table 1). The highest soluble substance content was found in the pomegranate peel extract (2.47 °Brix). No significant differences were found in the parameter values between BPE and GPE. Chemical compounds contained in the extracts, such as saponins, tannins, and polysaccharides, may affect the consistency and durability of cosmetics and interact with other ingredients in the formulation [48,49].

The highest polyphenol content was observed in the banana and pomegranate peel extracts. Noteworthy, the statistical analysis results indicated no significant difference in the polyphenol content between these two extracts. The overall content of these compounds ranged from 10.98 to 16.55 mg·100 mL^−1^. In the experiment conducted by Wasilewski et al. [45], higher polyphenol values were observed in grape pomace extract. The authors used a similar extraction method, but the by-product was frozen after pressing, whereas in our experiment, the pomace was dried by convection (50 °C, 8 h). These differences may indicate partial loss of polyphenols during drying and storage. It should also be noted that the by-product used by Wasilewski et al. [45] contained potassium metabisulfite, which, among other things, prevents polyphenol oxidation. Antioxidants are valuable compounds that disrupt radical chains and protect cells from damage caused by reactive species. Polyphenols, among others, are powerful antioxidants that protect human cells and tissues from oxidative stress. As demonstrated by Matos et al. [50], the presence of polyphenols in fruit pomace extracts contributes to their high antioxidant activity. Yarovaya and Khunkitti [51] reported that grape seed extract had better antioxidant activity than BHT (butylhydroxytoluene), which serves as an antioxidant and preservative in cosmetics [35,50,51].

The highest carotenoid content was observed in the extract obtained from tomato pomace. However, it is worth noting that quite large amounts of these components were also obtained from banana peels and grape pomace. Tomatoes and their residues are a valuable source of active compounds, including carotenoids [52]. Carotenoids are phytochemicals with antioxidant properties that interact synergistically with other antioxidants, protecting cells and tissues from oxidative damage [52]. Depending on the technique and extraction medium, the level of carotenoids recovered from tomato pomace may vary significantly. Extraction performed using SuperCritical-CO_2_ exhibited the highest carotenoid extraction rate (30 times higher than maceration with deep eutectic solvents) [53].

The vitamin C content in the extracts varied significantly. It should be noted that the highest concentration of this substance was observed in PPE. Opara et al. [54] reported that the vitamin C content in pomegranate peel may also vary significantly depending on the fruit variety. However, the researchers pointed out that all pomegranate varieties contained high amounts of vitamin C in the peel (~75–115 mg·100 g^−1^ fresh weight). According to the available literature data, grape or tomato pomace contain lower amounts of vitamin C than pomegranate peel (26–60 mg·100 g^−1^ dry weight) [55,56]. In turn, Wani [57] indicated that ripe banana peels may have higher amounts of antioxidants, such as vitamin C, than unripe ones. In the ripening phase, banana peel contains 6.9–10 mg of ascorbic acid·100 g^−1^. These data explain the significantly lower vitamin C content observed in the banana peel extracts analyzed in this study. By reducing oxidative stress caused by external and internal factors and by promoting the expression and maturation of collagen genes, vitamin C slows down the natural aging of the skin and weakens photoaging [58].

### 2.2. Characteristics of the Obtained Gels

**Figure 1:**
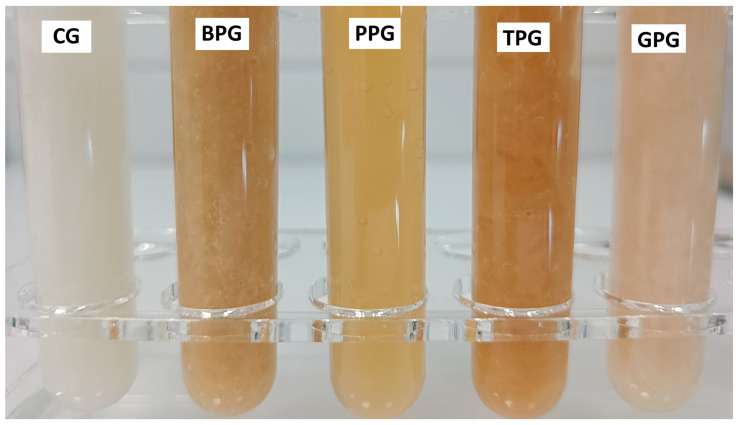
Prepared gels: CG—control gel, BPG—gel with banana peel extract, PPG—gel with pomegranate peel extract, TPG—gel with tomato pomace extract, GPG—gel with grape pomace extract.

The gels produced with the addition of extracts from by-products are shown in Figure 1.

The results of the solubility tests of the shower gels in distilled water are presented in Table 2. The gel with the pomegranate peel extract exhibited the best solubility in distilled water (15.7 min). A significantly longer solubility time was observed for samples containing the other plant extracts. However, it should be noted that the longest solubility time was observed for the control gel. Therefore, the addition of the plant extracts to the cleansing gel formula reduced the solubility time by almost 60%. The solubility of cleansing gels plays a crucial role in the effective cleansing of skin from all contaminants. The high solubility of these products significantly affects the effectiveness of the cleansing process, as it accelerates its course and facilitates rapid rinsing of the product from the skin [59].

The control gels as well as those enriched with the with banana peel extract and the grape pomace extract exhibited similar viscosity levels (402.9–412.1 mPa). The addition of the grape extract to the cosmetic resulted in an increase in viscosity to 416.8 mPa. Significantly lower viscosity was noted for the PPG and TPG samples. One commonly used method for increasing the viscosity of cleansing cosmetics is the use of hydrophilic rheology regulators. These are typically polymers containing numerous polar functional groups that readily hydrate. Xanthan gum used in the formulation is an example of a natural polymer fulfilling this function. According to Wasilewski et al. [45], the reduction in gel viscosity with the addition of extract containing a significant amount of bioactive compounds may be related to the presence of active ingredients that affect the gel-forming ability of thickeners in the formulation.

The density of the tested gels varied significantly depending on their composition. Gels with the pomegranate peel, tomato pomace, and grape pomace extracts were significantly denser than the control gel (up to 0.999 g·cm^−3^ for TPG). The gel with the banana peel extract had the lowest density (0.735 g·cm^−3^). The results suggest that the appropriate selection of ingredients influences the formulation density, which may be important for the product’s functional properties. According to research conducted by Gatti et al. [60], the preferred properties of a shower gel are a delicate fragrance and a sufficiently high density (package weight). Thanks to a sufficiently high density, a gel is perceived as more efficient in use.

The average pH values are presented in Table 2, along with the results of the Tukey test. For the prepared gels, the pH ranged from 5.391 to 5.917. The average pH values of the developed cosmetic formulations were within the normal pH range of human skin (between 4 and 6). Maintaining this acidity level supports the skin’s natural defense mechanisms against harmful microorganisms [61]. However, it should be noted that the addition of the extracts from the by-products resulted in a significant reduction in the pH of the gels. Fruit by-products contain organic acids responsible for the low pH. The impact of pH on skin condition is well illustrated in the study conducted by Kim et al. [62]. In an experiment involving 20 healthy individuals, products with pH 3, 6, and 8 were tested for five weeks. The results showed that the use of alkaline cosmetics (pH > 7) led to increased transepidermal water loss (TEWL). This means that the pH of skincare products has a significant impact on maintaining the skin’s hydrolipid barrier. The acidic pH of cosmetics used for daily hygiene plays a crucial role in maintaining a healthy skin microbiome, which is an integral part of its protective barrier. An increased epidermal pH can weaken the skin’s barrier function, which in turn results in greater permeability to microorganisms, allergens, and environmental pollutants. Additionally, it can cause excessive water loss from deeper layers of the skin, negatively impacting its hydration and overall condition [61,62].

The addition of the plant extracts to the washing gel formulation resulted in a significant increase in turbidity. The highest value of this parameter (12.2 NTU) was recorded for the gel containing tomato pomace. This may indicate a higher content of fine-particle components or greater aggregation of substances compared to the other samples. Some compounds in the extracts can destabilize the gel structure, leading to turbidity or even delamination. Furthermore, the extracts may contain natural particles that do not dissolve completely in water or the gel base [63,64].

**Figure 2:**
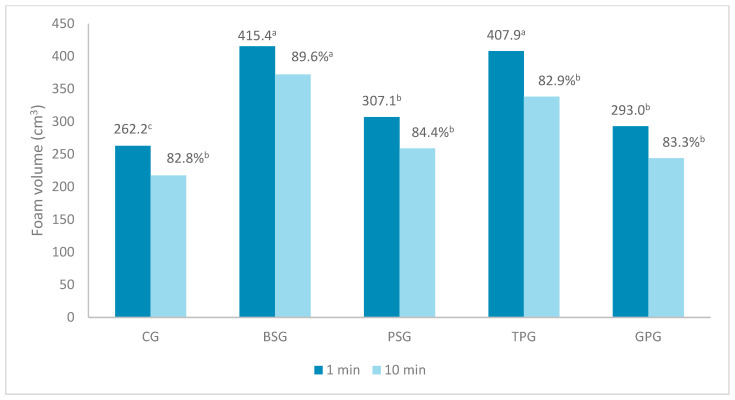
Foaming ability and foam stability (*FSI* is given in the graph as a percentage). Values of each parameter in a row with different superscript letters are significantly different (Tukey test. *p* ≤ 0.05).

Foam volume measured 1 and 10 min after formation is shown in Figure 2. The presence of the peel and pomace extracts was found to have a positive effect on the foaming capacity of the shower gels. The highest foam volume was recorded for the gel with the banana peel extract (415.4 cm^3^) and the gel with the tomato pomace extract (407.9 cm^3^). In the intended applications (cleansing gels), foaming capacity is an important characteristic influencing user ratings. The ability to produce an increased foam volume in gels enriched with plant extracts may result from the presence of natural saponins, polysaccharides, or surfactants that aid in foam stabilization. Banana peels are a source of saponins. Appropriate selection of the temperature and pH of the extraction medium allows effective extraction (41.75%). The banana peel biosurfactant exhibited strong surface-active properties, including foaming, emulsifying, and reduced surface tension [65]. The high foaming ability of TPG can be explained by the presence of furostanol saponins—a group of steroid glycosides characterized by the ability to generate foam in aqueous solutions. Furthermore, Wasilewski [45] explains that fruit pomace extracts stimulate foaming functions in cosmetics, even if the extract itself does not increase viscosity or foaming in model shower gels.

The highest foam stability was observed for the gel with the banana peel extract. The other samples were characterized by a similar, relatively high (>80%) foam stability. A formulation characterized by satisfactory usable quality produces long-lasting foam. Manufacturers pay particular attention to developing a cleansing base with appropriate foaming properties for the intended product. High foam stability is a particularly desirable feature in cleansing cosmetics, as foam formation is associated with both cleansing and relaxing effects [66].

Table 3 presents the color parameters of the gels. The highest L* parameter value, i.e., the lightest color, was recorded for the control gel (L* = 52.81). The modification of the gel formula resulted in a decrease in color brightness. The greatest changes were observed for the gel with the addition of the pomegranate peel extract (L* = 32.27).

The a* and b* color coordinates also differed significantly depending on the type of extract used in the cleansing gel formulas. The addition of each of the by-product extracts resulted in a significant increase in the a* parameter compared to the control sample. However, it should be noted that only for cosmetics containing the banana peel and tomato pomace extracts did the a* parameter achieve positive values, indicating an increased red color. Biocosmetics enriched with the by-product extract were also characterized by significantly greater yellow color intensity. The greatest changes were recorded for the sample containing the banana extract; the value of the b* parameter of this product was almost nine times higher than that of the control sample.

The total color change Δ*E* was also calculated in the experiment. The greatest color changes compared to the control sample were found after the addition of the pomegranate peel extract and the tomato pomace extract to the gel formulation. Noteworthy, for all modified gels tested, the total color change Δ*E* was above 5, indicating a significant color change (the observer could clearly distinguish two different colors).

A study by Zięba et al. [67] demonstrated that the inclusion of plant powders in cosmetics led to changes in all measured color parameters. Most notably, the authors noted a decrease in the L* value compared to the baseline sample. Chemically, plants are rich in compounds that can be used as coloring agents in the food or cosmetics industry. The same may apply to natural cleansing gels. Extracts obtained from by-products of the fruit and vegetable industry may be an interesting alternative to artificial cosmetic dyes [67,68].

## 3. Materials and Methods

### 3.1. Research Material

**Figure 3:**
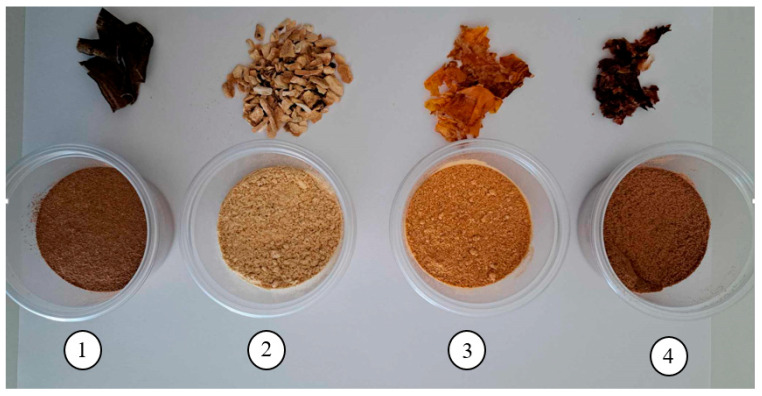
Dried fruit and vegetable by-products: before crushing (**top**) and after crushing (**bottom**): 1—banana peel, 2—pomegranate peel, 3—tomato pomace, 4—grape pomace.

The research material consisted of cleansing gels with the addition of extracts obtained from banana and pomegranate peels, as well as tomato and grape pomace (Figure 3). The obtained waste was dried by convection at 50 °C for 8 h using a laboratory dryer (POL-EKO SLN 15, Wodzisław, Poland) and ground using a laboratory grinder (Chemland FW100, Stargard, Poland).

### 3.2. Ultrasonic Extraction

The extraction was carried out using the method proposed by Wasilewski et al. [45] with minor modifications (ultrasound-assisted extraction, use of dried by-products). A 2% aqueous solution of decyl glucoside surfactant was used as the extraction medium. A 10 g portion of ground pomace was added to 190 g of the extraction medium (Table 4) and stirred at 380 rpm using a stirrer (MS 11 WIGO, Piastów, Poland) for 5 min. The extraction process was carried out using an ultrasonic bath (Elmasonic S60H, Singen, Germany) at a frequency of 37 kHz for 180 min. The process was conducted at room temperature. However, the temperature of the solution during the ultrasonic bath increased, reaching 55 °C in the final extraction phase. The extraction yield ranged from 16.2 mL/g to 17.6 mL/g. The obtained extract was filtered and used directly in further studies.

### 3.3. Determination of the Concentration of Soluble Substances

Total soluble substance content, expressed in °Brix, was determined by measuring the refractive index using a digital refractometer (LLG-uniREFRACTO, Meckenheim, Germany).

### 3.4. Determination of Polyphenol Content in the Extracts

Total polyphenol content was determined spectrophotometrically. Extract samples were diluted with 80% methanol in a 1:1 ratio (2 mL of each liquid). The mixture was then shaken for 30 min at 200 RPM (ELMI Orbital Shaker S-3.02.20M, Riga, Latvia) and centrifuged using an centrifuge (MPW-54, Warsaw, Poland). 100 µL of each extract solution was collected, and 2 mL of distilled water, 200 µL of Folin-Ciocalteau reagent (diluted 1:10), and 1 mL of 20% sodium carbonate solution were added. After 60 min at room temperature in the dark, total polyphenol content was determined using the Folin-Ciocalteau method [65]. Absorbance was read at λ = 765 nm on a Helios Omega UV-Vis spectrophotometer (Thermo Scientific, Waltham, MA, USA). A calibration curve was prepared using gallic acid. Results are expressed as mg of gallic acid per 100 mL of sample.

### 3.5. Carotenoid Content Determination

Total carotenoid content was determined according to the method described by Krajewska et al. [69]. The extract (0.5 g) was mixed with a mixture of acetone and 0.2% butylhydroxytoluene, and ethanol and hexane were added in a 1:1:2 ratio (25 mL). The absorbance of the hexane phase was measured using a Helios Omega 3 UV/Vis spectrophotometer (Thermo Scientific, Waltham, MA, USA) at a wavelength of 450 nm. Results were expressed in milligrams per 100 mL of extract.

### 3.6. Vitamin C Content Determination

Vitamin C content in the tested extracts was determined using the iodometric method described by Krajewska et al. [69]. 10 mL of the tested extract was titrated with a standard iodine solution until a dark blue color was obtained (in the presence of a 1% starch solution). The titer of the iodine solution was determined by titrating 10 cm^3^ of an L-ascorbic acid solution of known concentration. Results were expressed in milligrams per 100 mL of extract.

### 3.7. Production of Cleansing Gels

The raw materials used and their quantities are presented in Table 5. The appropriate extracts were added to the previously weighed raw materials. The mixture was placed on a mixer (MS 11 WIGO, Piastów, Poland)) and mixed for 2 min. Mixing was then carried out using a homogenizer (CAT, Unidrive 1000D, Ballrechten-Dottingen, Germany) for 5 min. The prepared gels (Figure 1) were transferred to containers, tightly closed, and stored at refrigerated temperature.

### 3.8. pH Analysis

A total of 1 g of each preparation was dispersed in 100 mL of distilled water (pH 6.98). The pH was measured in triplicate using a digital pH meter (Metrohm 801, Herisau, Switzerland).

### 3.9. Calculation of the Density of Each Gel

Exactly 2 mL of each gel was collected and weighed after application to the slide. The test was repeated three times. The density of each gel was calculated as the quotient of mass (g)/volume (2 cm^3^).

### 3.10. Turbidity Determination

For the determination, a 4% aqueous solution of the preparation was prepared. The test was performed with the nephelometric method using a turbidimeter (HACH TL2310, Loveland, CO, USA).

### 3.11. Water Solubility Testing

A total of 100 mL of distilled water was poured into a 150 mL beaker and placed on a magnetic stirrer set to 100 rpm. Using a syringe, 2 mL of the tested gel was injected into the water. Each sample was then stirred until the gel was completely dissolved in the water. Measurements were taken three times at 23 °C.

### 3.12. Viscosity Testing

The test was conducted using the back extrusion method. This method involves forcing a 46 mm diameter cylindrical head through a sample placed in a 48 mm diameter cylindrical container and measuring the force required to move the head by 20 mm. A Zwick/Roel Z0.5 (Ulm, Germany) tensile testing machine was used. The test was performed three times in 5 cycles, at speeds of 50, 100, 200, 400, and 800 mm/min at a temperature of 23 °C. The average dynamic viscosity (mPa) was calculated using testXpert II (V3.5) software.

### 3.13. Foaming Capacity and Foam Stability Index Test

For this test, a 4% aqueous solution of the preparation was prepared according to the Ross-Miles method, in accordance with Polish Standard PN-ISO 696:1994 [70]. A 5 mL portion of the solution was poured into a cylinder, and a 500 mL portion of the solution was poured into a separatory funnel, which was placed 45 cm above the liquid level in the cylinder, and the dropper tap was turned on. The foam height formed in the cylinder was measured 1 min after formation and 10 min later. Based on these measurements, the foaming capacity was determined.

The foam stability index (*FSI*) was calculated as a percentage using Formula (1):

(1) $$FSI=\frac{h_{2}}{h_{1}}\cdot 100\%,$$

where *h*_1_—foam height measured immediately after 1 min (cm^3^); *h*_2_—foam height measured after 10 min (cm^3^).

### 3.14. Measurement of Color Parameters

The color of the samples was measured with the 3Color SF80 spectrophotometer (Narama, Poland), using a D65° illuminator at an observation angle of 10°. The CIE Lab color scale was used for analysis: L* (lightness/darkness), a* (red/greenness), and b* (yellowness/blueness). The total color change Δ*E* was calculated using Formula (2) [45]:

(2) $$∆E=\sqrt{{\left(∆L\right)}^{2}+{\left(∆a\right)}^{2}+{\left(∆b\right)}^{2}}$$

where Δ*L*, Δ*a*, and Δ*b* denote the difference in the color of the gel samples with the addition of one of the by-product extracts compared to the control gel.

### 3.15. Statistical Analysis

Data were analyzed using the Statistica software package (version 13, StatSoft Inc., Tulsa, OK, USA). Analysis of variance was performed using ANOVA (StatSoft Inc., Tulsa, OK, USA) (Tukey’s test).

## 4. Conclusions

Incorporating extracts from plant by-products into a cleansing gel formula creates a cosmetic product containing natural polyphenols and characterized by distinctive color, greater foaming capacity, lower pH, and shorter dissolving time. The addition of plant extracts significantly influences the color of finished cosmetic products—both in terms of brightness (adding the extract darkens the cleansing gel) and the color tone of the final product—shifting its chromaticity toward red and yellow. Therefore, extracts from by-products can be an alternative to artificial colors used in cosmetics.

Extracts from plant by-products have been shown to contain natural polyphenols, carotenoids, and vitamin C. Given the protective properties and ability of these compounds to neutralize free radicals, these extracts can be valuable cosmetic ingredients. Banana peel and pomegranate extracts stand out for their high polyphenol content (among all the raw materials included in the research program). Pomegranate peel extract also contained the highest amount of vitamin C. Tomato pomace was the best source of carotenoids among the recycled raw materials tested. The addition of fruit and vegetable peel and pomace extracts significantly improved the foaming properties of cleansing gels, which is crucial for their functionality. The present results indicate the potential of natural extracts to improve foam quantity and stability. All developed cleansing gels have a pH within the physiological, slightly acidic range of 5.39–5.92, consistent with the natural pH of human skin. Maintaining this pH is crucial for the integrity of the skin’s protective barrier, proper hydration, and a healthy microbiome. The density of cosmetics containing pomegranate peel extract and tomato and grape pomace was higher than that of the control product. The ingredients used in the formulation have a significant impact on the physical properties of the product, and a sufficiently high gel density can positively impact consumer perception and perceived effectiveness during use. The turbidity of cleansing gels increases after the addition of fruit and vegetable by-product extracts to the formula. Shower gels containing by-product extracts demonstrate better solubility in distilled water compared to the control gel, confirming their improved effectiveness in cleansing skin and greater comfort for consumers. The addition of pomegranate peel extracts to the cleansing gel formula results in a reduction in the viscosity of the formulation.

In summary, extracts obtained from fruit and vegetable processing can positively impact the physicochemical and functional properties of cleansing gels. Their use contributes to increased antioxidant potential, improved foaming capacity, and improved solubility, while maintaining a pH suitable for the skin. In addition to improving functional properties, obtaining extracts from by-products supports the concept of a circular economy and reduces the amount of industrial waste. Furthermore, incorporating extracts from fruit and vegetable residues into cleansing gel formulations can contribute to a reduction in the amount of added colorants and pH-regulating substances. To ensure the efficacy and safety of the commercial use of these plant extracts, further studies are necessary. In particular, analyses aimed at determining the microbiological stability and the irritation potential of the obtained cleansing gels are required. Attention should also be paid to ensuring acceptable sensory quality. The present results support the need for further research into the use of by-products from the fruit and vegetable industry in the cosmetics industry, particularly with regard to the stability and sensory properties of sustainable cosmetic formulations.

## Figures and Tables

**Table 1:** Contents of soluble solids, vitamin C, carotenoids, and polyphenols in extracts from by-products.

Probe	SSC (°Brix)	TPC (mg·100 mL^−1^)	TCC (mg·mL^−1^)	Vitamin C (mg·100 mL^−1^)
BPE	2.30 ± 0.00 ^b^	16.54 ± 0.03 ^a^	1.831 ± 0.085 ^b^	0.651 ± 0.056 ^c^
PPE	2.47 ± 0.06 ^a^	16.52 ± 0.04 ^a^	1.391 ± 0.049 ^c^	1.529 ± 0.056 ^a^
TPE	2.13 ± 0.06 ^c^	10.99 ± 0.04 ^c^	2.402 ± 0.028 ^a^	1.139 ± 0.056 ^b^
GPE	2.33 ± 0.06 ^b^	13.44 ± 0.05 ^b^	1.831 ± 0.085 ^b^	1.236 ± 0.113 ^b^

BPE—banana peel extract, PPE—pomegranate peel extract, TPE—tomato pomace extract, GPE—grape pomace extract; SSC—soluble solids content, TPC—total phenolic content, TCC—total carotenoid content. Values of each parameter in a row with different superscript letters are significantly different (Tukey test. *p* ≤ 0.05).

**Table 2:** Characteristics of the obtained gels with the addition of extracts from by-products.

Probe	Solubility (min)	Viscosity (mPa)	Density (g·cm^−3^)	pH (-)	Turbidity (NTU)
CG	39.0 ± 2.6 ^a^	402.9 ± 2.9 ^b^	0.856 ± 0.004 ^c^	5.917 ± 0.002 ^a^	8.98 ± 0.07 ^c^
BPG	27.7 ± 1.5 ^b^	412.1 ± 5.0 ^ab^	0.735 ± 0.005 ^d^	5.594 ± 0.003 ^d^	11.47 ± 0.23 ^ab^
PPG	15.7 ± 1.5 ^c^	303.4 ± 3.1 ^d^	0.974 ± 0.010 ^b^	5.786 ± 0.021 ^b^	10.67 ± 0.15 ^b^
TPG	29.0 ± 1.0 ^b^	358.8 ± 7.6 ^c^	0.996 ± 0.002 ^a^	5.391 ± 0.005 ^e^	12.23 ± 0.40 ^a^
GPG	29.7 ± 0.6 ^b^	416.8 ± 4.0 ^a^	0.999 ± 0.001 ^a^	5.723 ± 0.009 ^c^	10.80 ± 0.50 ^b^

CG—control gel, BPG—gel with banana peel extract, PPG—gel with pomegranate peel extract, TPG—gel with tomato pomace extract, GPG—gel with grape pomace extract. Values of each parameter in a row with different superscript letters are significantly different (Tukey test. *p* ≤ 0.05).

**Table 3:** Color parameters of the gels with the addition of extracts from by-products.

Probe	L*	a*	b*	Δ*E*
CG	52.81 ± 0.52 ^a^	−0.89 ± 0.03 ^e^	1.71 ± 0.13 ^d^	0
BPG	46.31 ± 0.32 ^b^	1.46 ± 0.08 ^a^	15.03 ± 0.41 ^a^	15.01
PPG	32.27 ± 0.25 ^e^	−0.61 ± 0.03 ^d^	3.89 ± 0.07 ^c^	20.65
TPG	34.88 ± 1.20 ^d^	1.20 ± 0.20 ^b^	9.36 ± 1.03 ^b^	19.61
GPG	38.40 ± 0.83 ^c^	−0.40 ± 0.05 ^c^	3.43 ± 0.16 ^c^	14.52

CG—control gel, BPG—gel with banana peel extract, PPG—gel with pomegranate peel extract, TPG—gel with tomato pomace extract, GPG—gel with grape pomace extract. Values of each parameter in a row with different superscript letters are significantly different (Tukey test. *p* ≤ 0.05).

**Table 4:** Composition used to prepare the peel and pomace extracts.

Ingredients (g)	By-Product Extract				
	CE	BPE	PPE	TPE	GPE
Distilled water	185	185	185	185	185
Decyl Glucoside	4	4	4	4	4
DHA-BA	1	1	1	1	1
Dry by-products	-	Banana peel	Pomegranate peel	Tomato pomace	Grape pomace
		10	10	10	10

CE—control eCE—control extract, BPE—banana peel extract, PPE—pomegranate peel extract, TPE—tomato pomace extract, GPE—grape pomace extract. DHA BA—A blend of preservatives approved by Eco-cert for use in organic cosmetics. INCI (International Nomenclature of Cosmetic Ingredients): Dehydroacetic Acid, Benzyl Alcohol.

**Table 5:** Recipe for cleansing gels with the addition of extracts from by-products.

Ingredients (%)	Control Extract	Banana Peel Extract	Pomegranate Peel Extract	Tomato Pomace Extract	Grape Pomace Extract	DHA BA	SCI	Coconut Betaine	Sodium Chloride	Distilled Water	Decyl Glucoside	Xanthan Gum
CG	65	0	0	0	0	1	4.5	2	1	Up to 100	4	1.4
BPG	0	65	0	0	0	1	4.5	2	1	Up to 100	4	1.4
PPG	0	0	65	0	0	1	4.5	2	1	Up to 100	4	1.4
TPG	0	0	0	65	0	1	4.5	2	1	Up to 100	4	1.4
GPG	0	0	0	0	65	1	4.5	2	1	Up to 100	4	1.4

CG—control gel, BPG—gel with banana peel extract, PPG—gel with pomegranate peel extract, TPG—gel with tomato pomace extract, GPG—gel with grape pomace extract.

## Data Availability

Data are contained within the article.
